# Camera, LiDAR, and IMU Spatiotemporal Calibration: Methodological Review and Research Perspectives

**DOI:** 10.3390/s25175409

**Published:** 2025-09-02

**Authors:** Xinyu Lyu, Songlin Liu, Rongcan Qiao, Songyang Jiang, Yuanshi Wang

**Affiliations:** 1 School of Computer, Qufu Normal University, Rizhao 276800, China; andylv686@qufu.edu.cn (X.L.); qrc20010515@163.com (R.Q.); jiangsongyang@qfnu.edu.cn (S.J.); ys_wang@qfnu.edu.cn (Y.W.); 2 Fifth Faculty, Information Engineering University China, Zhengzhou 450013, China

**Keywords:** multi-sensor calibration, camera, LiDAR, IMU

## Abstract

Multi-sensor fusion systems involving Light Detection and Ranging (LiDAR), cameras, and inertial measurement units (IMUs) have been widely adopted in fields such as autonomous driving and robotics due to their complementary perception capabilities. This widespread application has led to a growing demand for accurate sensor calibration. Although numerous calibration methods have been proposed in recent years for various sensor combinations, such as camera–IMU, LiDAR–IMU, camera–LiDAR, and camera–LiDAR–IMU, there remains a lack of systematic reviews and comparative analyses of these approaches. This paper focuses on extrinsic calibration techniques for LiDAR, cameras, and IMU, providing a comprehensive review of the latest developments across the four types of sensor combinations. We further analyze the strengths and limitations of existing methods, summarize the evaluation criteria for calibration, and outline potential future research directions for the benefit of the academic community.

## 1. Introduction

In recent years, advancements in sensor technology and the substantial growth in computing power have driven increasing interest in multi-sensor fusion for applications such as autonomous driving [1], robotic navigation, simultaneous localization and mapping (SLAM) [2,3], and intelligent transportation systems [4]. Among these, multi-sensor fusion has become a core strategy in autonomous driving, supporting precise localization and comprehensive perception of complex environments. Representative applications include autonomous mining trucks, port transportation vehicles, and driverless taxis.

By leveraging the complementary capabilities of heterogeneous sensors such as cameras, Light Detection and Ranging (LiDAR), and inertial measurement units (IMUs), researchers have developed multimodal perception frameworks that significantly enhance system robustness and scene understanding. However, the effectiveness of data fusion depends fundamentally on accurate spatiotemporal calibration. Extrinsic calibration determines the rigid body transformation (rotation and translation) between sensor coordinate frames, aligning heterogeneous data within a unified reference frame and eliminating spatial mismatches due to frame discrepancies. Even small calibration errors can lead to great misalignment in cross-modal data fusion.

Among various configurations, systems combining cameras, LiDAR, and IMUs are widely adopted [5]. Cameras provide dense two-dimensional pixel arrays with rich texture and semantic information but are susceptible to lighting conditions and motion blur. LiDAR emits laser pulses to generate three-dimensional point clouds, offering centimeter-level spatial accuracy and stable performance under challenging lighting. However, LiDAR typically operates at lower frequencies and produces sparse point clouds with limited detail for small or distant objects. Moreover, it lacks semantic content. IMUs measure angular velocity and linear acceleration at high frequencies but are prone to integration drift caused by sensor bias and noise.

In multimodal systems that integrate a camera, a LiDAR, and an IMU, intrinsic calibration is typically performed first, followed by extrinsic calibration between two selected sensors, including the camera–IMU, LiDAR–IMU, and camera–LiDAR pairs. Intrinsic calibration methods for individual sensors are well established, as reviewed in [6,7,8,9,10,11]. In recent years, significant efforts have been devoted to the calibration of sensor pairs, such as camera–IMU, LiDAR–IMU, and camera–LiDAR. However, relatively few studies have explored the calibration of systems involving three sensors: camera, LiDAR, and IMU. Furthermore, a systematic review of joint calibration methods for camera–IMU and LiDAR–IMU pairs remains lacking. By contrast, due to their widespread application, the fusion and calibration of camera and LiDAR have been thoroughly investigated. This disparity in research efforts underscores the need for a comprehensive survey dedicated to the joint extrinsic calibration for camera, LiDAR, and IMU. The main contributions of this review are as follows:

- This paper presents a comprehensive overview of sensor calibration methods, beginning with the fundamental principles of individual sensors in order to construct measurement models and to introduce intrinsic calibration techniques. To the best of our knowledge, it is the first paper to systematically review and analyze extrinsic calibration approaches for three sensor pairings (camera–IMU, LiDAR–IMU, and their joint configuration), addressing a gap in existing literature on multimodal sensor calibration.
- This paper summarizes the classification of multi-sensor calibration methods and key factors for evaluating calibration performance. It also provides an overview of open-source calibration tools, detailing their technical characteristics and applicable sensor types. These resources help to reduce redundant development efforts for researchers. Finally, the paper outlines potential future research directions to serve as a reference for continued exploration.

We provide a detailed overview of multi-sensor calibration systems through five main sections. Section 1 serves as the introduction, outlining the scope of this work. Section 2 discusses the fundamental principles and mathematical models of the three sensors, along with a brief summary of intrinsic calibration and temporal offset calibration. Section 3 presents the technical approaches for extrinsic calibration of camera–IMU, LiDAR–IMU, camera–LiDAR, and camera–LiDAR–IMU systems. Section 4 examines key factors for evaluating calibration methods, summarizes several open-source methods and datasets for camera–LiDAR–IMU calibration, and highlights potential research directions. Finally, Section 5 concludes the paper.

## 2. Camera–LiDAR–IMU Calibration System

In this section, we first introduce the fundamental classification of camera–LiDAR–IMU integrated systems, followed by an explanation of the operating principles of each sensor. Finally, we briefly outline the intrinsic calibration methods for individual sensors, which serves as a necessary prerequisite for spatiotemporal extrinsic calibration in multi-sensor systems.

### 2.1. Classification

**Figure 1:**
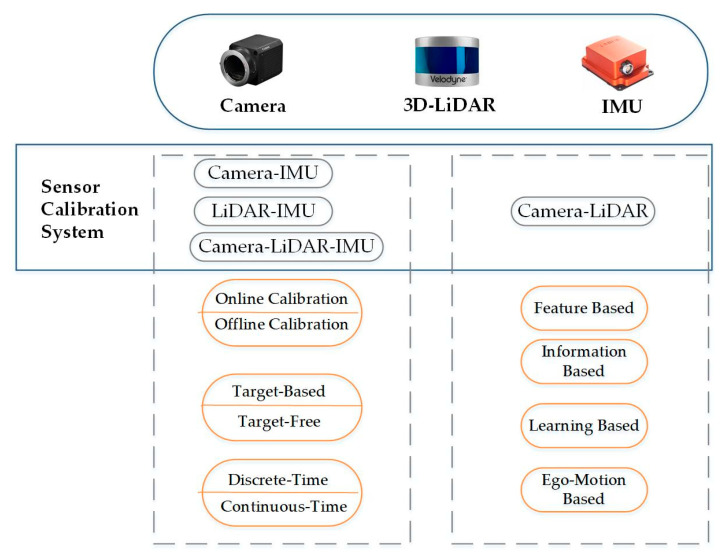
Classification of the Camera–LiDAR–IMU Calibration System.

In camera–LiDAR–IMU systems, four typical sensor combinations are commonly considered: camera–IMU, LiDAR–IMU, camera–LiDAR, and the full integration of camera–LiDAR–IMU, as illustrated in Figure 1. Calibration methods for these systems can be classified in several ways. One common classification is based on whether artificial targets are used, which leads to two main categories: target-based calibration and targetless calibration.

**Target-based methods:** These methods establish cross-sensor data associations by introducing specifically designed calibration objects. Common examples include checkerboards or AprilTags [12] for cameras, geometric feature boards for LiDAR, and corner reflector arrays for radar. These methods take advantage of well-defined geometric structures, which simplify data association and help ensure calibration accuracy. However, they require a static environment and are unsuitable for real-time calibration in dynamic settings.

**Targetless methods:** These methods extract features directly from natural environments, such as SIFT [13] or SURF [14] keypoints, lines, or planar surfaces, and leverage motion consistency constraints from IMU measurements to establish data association. Therefore, their performance largely depends on the number of features in natural scenes.

From the perspective of the processing mode, calibration methods are categorized as either offline or online. Online calibration continuously updates calibration parameters by integrating them into a joint state estimation framework, where they are optimized alongside other states. In contrast, offline calibration determines the calibration parameters in a single batch once data collection has been completed.

In terms of temporal modeling, calibration approaches are divided into a discrete-time and continuous-time model based on how system states are represented. Traditional methods typically adopt discrete-time models that solve for states at sampled time steps. In high-dynamic motion scenes, these approaches often require interpolating asynchronous sensor measurements, such as aligning timestamps, which can introduce errors and reduce accuracy. The continuous-time model treats the system trajectory as a continuous function, such as a polynomial basis or B-spline, allowing for precise the modeling of motion under non-uniform sampling or rapid movement. However, this increases computational complexity and imposes greater demands on hardware.

Specifically, camera–LiDAR calibration methods can be grouped into four categories: feature-based methods, information-based methods, motion-based methods, and learning-based methods. Section 3.3 provides a detailed discussion of these categories.

### 2.2. Fundamental Principles of Individual Sensors

#### 2.2.1. Camera

As a widely used imaging sensor, the camera operates based on the principles of optical imaging. Light from the external environment passes through the camera’s lens system, where it is refracted and focused onto a pixel array within the image sensor, such as a CMOS or CCD. Through photoelectric conversion, the optical signal is transformed into a digital image. This imaging process is governed by the camera’s intrinsic parameters, including focal length, principal point, and lens distortion, which together define the projection relationship from the three-dimensional world to the two-dimensional image plane. In addition to monocular cameras, stereo and depth cameras are also commonly used. Traditional monocular cameras have more limitations than stereo and depth cameras because they do not inherently provide depth information. Depth must be inferred through triangulation, which restricts their adaptability in complex or dynamic environments.

The depth estimation principle of a stereo camera relies on capturing images of the same scene from two slightly different viewpoints using two cameras. By leveraging the concept of disparity and applying triangulation, the distance between the object and the camera can be computed. For example, when the baseline distance is known, the target distance can be directly derived using focal length and disparity values through Equation (1). The depth perception range of a binocular camera extends with the baseline length, where a longer baseline can cover distances of several tens of meters. However, since binocular cameras rely on disparity matching computations, they require highly precise hardware calibration. Factors such as lens optical axis alignment and pixel synchronization errors can significantly affect the reliability of disparity calculations. Equation (1) is formulated as follows:

(1) $$Z=\frac{f\cdot b}{d}$$

where *Z* denotes the depth, *f* the focal length, *b* the baseline, and *d* the disparity.

Depth cameras directly obtain the depth information of the target. Based on the method of acquiring depth values, they can be categorized into time of flight (ToF) and structured light approaches. ToF methods measure the distance by emitting light pulses towards the target and calculating the time difference between emission and reflection. Structured light methods involve projecting a known light pattern (e.g., Gray code or random speckles) onto the target and computing the distance based on the deformation of the pattern upon contact with the object. Depth cameras offer millimeter-level accuracy but have a limited effective range, making them primarily suitable for close-range indoor environments.

#### 2.2.2. LiDAR

LiDAR is a sensing technology that determines the distance between a device and a target based on either the ToF or the phase shift of emitted light pulses. LiDAR sensors output data as a set of spatial points, known as point cloud data (PCD), which include the x, y, and z coordinates along with intensity values reflecting the strength of the returns from objects or obstacles in the surrounding environment.

Based on their scanning mechanisms, LiDAR systems can be broadly categorized into three types [15]: mechanical LiDAR, scanning solid-state LiDAR, and flash LiDAR with non-scanning architectures. The most common mechanical LiDAR uses rotating components to steer laser beams across various angles to achieve a wide field of view. In contrast, flash LiDAR eliminates all mechanical components by emitting a broad laser pulse that covers the entire field of view (FoV) in a single shot, while a focal plane array detector simultaneously captures the reflected signals from the scene, directly generating a three-dimensional point cloud.

Scanning solid-state LiDAR systems remove the need for mechanical rotation through various beam-steering techniques, such as micro-electro-mechanical systems (MEMSs) micromirrors, rotating mirrors, or prisms. These methods control the deflection of the laser beam over a limited angular range via electrical signals. By significantly reducing the size and complexity of the device while maintaining high accuracy, scanning solid-state LiDAR has become a mainstream solution in modern autonomous driving applications.

Although mechanical LiDAR systems have demonstrated reliable measurement performance, they are constrained by the limitations inherent to their mechanical components. Over time, rotating parts are prone to wear and require periodic maintenance to ensure sustained performance. Additionally, mechanical LiDAR units are susceptible to displacement and structural deformation under external forces such as vibrations or shocks, which can degrade scanning accuracy and system stability. In contrast, scanning solid-state and flash LiDAR systems face constraints primarily in terms of FoV. Mechanical LiDAR can readily achieve a full 360° horizontal FoV through continuous rotation. Scanning solid-state LiDAR can increase coverage via oscillating mirrors, but flash LiDAR are typically restricted to a fixed FoV.

#### 2.2.3. IMU

An Inertial Measurement Unit (IMU) typically consists of an accelerometer and a gyroscope, with some variants also including a magnetometer. By measuring angular velocity and linear acceleration, then performing integration, the system can estimate its position, velocity, and orientation. Unlike environment-dependent sensors such as cameras or LiDAR, the IMU is a self-contained localization sensor that operates in both indoor and outdoor settings and remains robust under poor lighting and dynamic scenes. However, IMU measurements are affected by various error sources, including bias drift, temperature-induced variations, sensor noise, and random walk. These errors accumulate during integration, leading to unbounded drift in the estimated state over time.

### 2.3. Intrinsic Calibration

Intrinsic calibration aims to estimate the geometric and physical parameters of individual sensors, such as the focal length and principal point offset of a camera, the bias of accelerometers and gyroscopes in an IMU, and distance correction parameters in a LiDAR sensor. These parameters are used to correct systematic or deterministic errors and to ensure the accuracy of data from each sensor. Once determined, intrinsic parameters are generally assumed to remain stable under ideal conditions. Algorithms and techniques for intrinsic sensor calibration have received considerable attention in recent years and have achieved significant progress. This subsection briefly outlines the core principles and provides references to the relevant review literature.

#### 2.3.1. Camera Model and Image Undistortion

**Figure 2:**
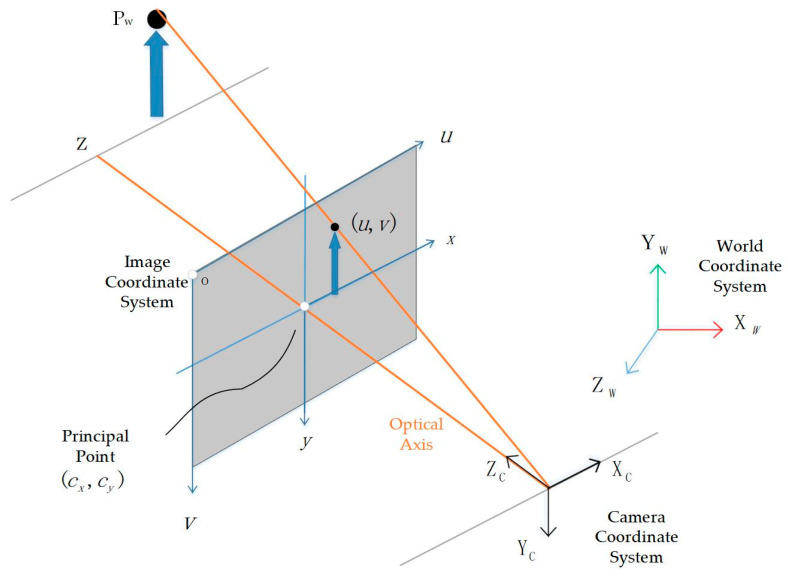
The pinhole camera model from a mathematical perspective. Figure redrawn based on depictions in [16].

Camera models have been well established, including the pinhole camera model, fisheye camera model, and omnidirectional camera model. Here, we take the pinhole model as an example, as illustrated in Figure 2. Under ideal conditions, a three-dimensional point in space can be projected onto the image plane according to Equation (2). Specifically, a point $P_{w}$ in the world coordinate system is transformed into the camera coordinate system as $P_{C}$ through the extrinsic transformation $T_{W}^{C}$, then projected onto the image plane as pixel coordinates P using the camera’s intrinsic matrix K:

(2) $$P=KP_{C}=KT_{W}^{C}P_{w}$$

where $P,P_{C},P_{w}$ denote the pixel coordinates, the coordinates in the camera coordinate system, and the coordinates in the world coordinate system, respectively. K is the intrinsic matrix of the pinhole camera, which encodes the camera’s geometric parameters, such as the skew coefficient (s), the principal point offsets $\left(c_{x},c_{y}\right)$, and the focal lengths $\left(f_{x},f_{y}\right)$. The transformation matrix $T_{W}^{C}$ represents the extrinsic parameters, which convert the three-dimensional point $P_{w}$ in the world coordinate system to the corresponding point in the camera coordinate system.

**Figure 3:**
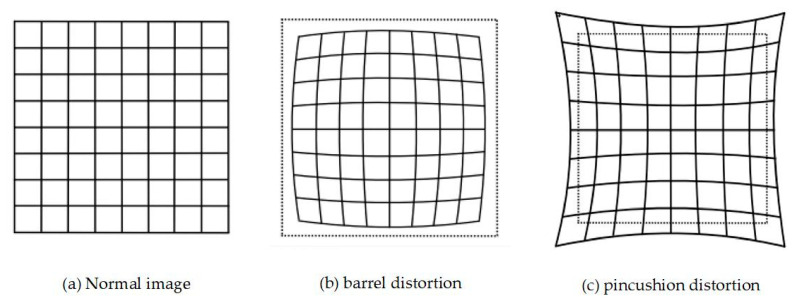
Two different types of radial distortion. In barrel distortion and pincushion distortion, the dashed lines indicate the size of a normal image.

In practice, light entering a camera is subject to radial distortion due to the lens’s imperfect curvature, while tangential distortion results from minor misalignments between the lens and the image plane during assembly. Radial distortion increases with distance from the image center and is more significant near the image boundaries. Radial distortion can be further classified into barrel distortion and pincushion distortion, as illustrated in Figure 3. The mapping between distorted and undistorted image points can be effectively modeled using polynomial functions:

(3) $$\left\{\begin{array}{c} \Delta x_{r}=\;x\;\cdot \left(k_{1}r^{2}\;+\;k_{2}r^{4}\;+\;k_{3}r^{6}\right)\\ \Delta y_{r}\;=\;y\;\cdot \left(k_{1}r^{2}\;+\;k_{2}r^{4}\;+\;k_{3}r^{6}\right) \end{array}\right.$$

where (x,y) denotes the ideal undistorted normalized image coordinates, $\left(\Delta x_{r},\Delta y_{r}\right)$ represents the coordinate deviations caused by radial distortion, $k_{1},\;k_{2}$ and $k_{3}$ are the radial distortion coefficients, and r is the Euclidean distance from the point (x,y) to the origin of the coordinate system.

Tangential distortion leads to pixel displacements along both radial and tangential directions. This type of distortion can be compensated by introducing two additional parameters, *p*_1_ and *p*_2_, as shown in Equation (4):

(4) $$\left\{\begin{array}{c} \Delta x_{t}\;=\;2p_{1}xy\;+\;p_{2}\left(r^{2}+2x^{2}\right)\\ \Delta y_{t}\;=\;p_{1}\left(r^{2}+2y^{2}\right)\;+\;2p_{2}xy \end{array}\right.$$

where $p_{1}$ and $p_{2}$ are the tangential distortion coefficients, and $\left(\Delta x_{t},\Delta y_{t}\right)$ represent the coordinate deviations caused by tangential distortion.

Finally, by combining radial and tangential distortions, the distorted coordinates $\left(x^{\prime },y^{\prime }\right)$ can be obtained, as shown in Equation (5):

(5) $$\left\{\begin{array}{c} x^{\prime }\;=\;x\;+\;\Delta x_{r}\;+\;\Delta x_{t}\\ y^{\prime }\;=\;y\;+\;\Delta y_{r}\;+\;\Delta y_{t} \end{array}\right.$$

In the preceding discussion, the imaging process was modeled using the pinhole camera model, incorporating radial and tangential distortions resulting from lens imperfections. In practical imaging systems, a variety of alternative models have been proposed, including the affine model [17] and perspective model, as well as other forms of distortion. Nonetheless, the combination of the pinhole model with radial and tangential distortion remains sufficiently accurate for the majority of applications.

A substantial body of research has focused on distortion calibration techniques across different camera types. Existing methods have been systematically reviewed and summarized to facilitate a comprehensive and accessible understanding of distortion calibration strategies. Hughes et al. [18] published the first review on camera distortion, summarizing calibration methods for various types of distortion in fisheye cameras, including radial distortion, uneven illumination, and inaccuracies in distortion center estimation. Ref. [19] further details distortion calibration techniques for wide-angle and fisheye cameras used in the automotive industry. Qi et al. [20] refined barrel distortion calibration methods by categorizing them into three types: affine transformation, quadratic polynomial transformation, and polar coordinate transformation. With the progress of calibration techniques, zoom lens calibration has received increasing attention. Ayaz et al. [21] reviewed zoom lens calibration methods and proposed a new classification scheme for monocular and stereo camera zoom lens calibration. They also provided a comparative analysis of the advantages and disadvantages of various methods. With the introduction of deep learning, distortion calibration has achieved significant progress. 

#### 2.3.2. LiDAR Measurement Model

The LiDAR measurement model follows the range–azimuth–elevation paradigm. A point P in the LiDAR coordinate system is defined by Equation (6):

(6) $$\left[\begin{array}{c} x\\ y\\ z \end{array}\right]=\left[\begin{array}{c} \mathrm{rcos}\alpha \cos\beta \\ \mathrm{rsin}\alpha \cos\beta \\ \mathrm{rsin}\beta \end{array}\right]$$

where r represents the distance from point P to the LiDAR, which is computed as the product of the light speed and half of the laser beam’s round-trip time. β denotes the azimuth angle and α denotes the elevation angle.

LiDAR measurement data are affected by systematic errors stemming from the sensor’s measurement principles and the precision of hardware assembly. These errors can be mitigated through LiDAR calibration procedures. The most common types of systematic errors include angular errors and range errors. Angular errors occur when the initial scanning angle deviates from its ideal orientation, typically due to manufacturing tolerances and assembly imperfections. Since the scanning mechanism involves rotating components, such as laser heads or prisms, to cover a predefined angular field, inaccuracies in the initial angle may propagate throughout the scan. This leads to pointing deviations, instantaneous angular discrepancies, and scan plane distortion. Range errors, on the other hand, primarily arise from issues during signal processing. These include misalignments between the transmitted and received laser beams, inaccuracies in ToF estimation, and mechanical vibrations affecting the sensor during operation. Systematic calibration is generally conducted by the manufacturer before the LiDAR system is shipped. As a result, end users typically do not need to perform manual compensation for these errors during normal use.

#### 2.3.3. Inertial Measurement Model

Unlike external imaging sensors, an IMU directly outputs raw measurements of acceleration and angular velocity. The measurement model for the three-axis accelerometer and gyroscope can be formulated as shown in Equation (7):

(7) $$v=\left[\begin{array}{c} v_{1}\\ v_{2}\\ v_{3} \end{array}\right]=\left[\begin{array}{c} k_{11}\;\;\;\;k_{12}\;\;\;\;k_{13}\\ k_{21}\;\;\;\;k_{22}\;\;\;\;k_{23}\\ k_{31}\;\;\;\;k_{32}\;\;\;\;k_{33} \end{array}\right]\left[\begin{array}{c} u_{1}\\ u_{2}\\ u_{3} \end{array}\right]+\left[\begin{array}{c} b_{1}\\ b_{2}\\ b_{3} \end{array}\right]=Ku+b$$

where u denotes the raw measurements, v represents the calibrated output, b is the bias error, and K is the scale factor matrix, which captures proportional errors, including scale factor deviations, cross-axis sensitivity, and sensor misalignment.

The errors in accelerometers and gyroscopes can generally be classified into deterministic errors and stochastic errors [22]. Deterministic errors typically originate from inaccurate factory calibration (or from incorrect conversion parameters specified in the sensor datasheet) [23], and usually include the following three types:

- Bias (Zero Offset): Bias error refers to a non-zero sensor output when no external stimulus is present. It is typically defined as the mean output of the accelerometer or gyroscope over a specified duration under static conditions.
- Scale Factor Error: The scale factor error describes the deviation between the actual sensor response and the ideal linear relationship between input and output. It is usually determined as the slope of the best-fit line derived from a set of input–output measurements.
- Non-Orthogonality and Misalignment Errors: Non-orthogonality errors arise from manufacturing imperfections that cause the sensing axes to deviate from perfect perpendicularity. Misalignment errors occur when the sensor’s sensitive axes are not precisely aligned with the axes of the mounting platform. These errors are influenced by the accuracy of the alignment between the sensor’s reference frame and the system coordinate frame.

Some errors or noise sources originate from random fluctuations in the system response and are classified as stochastic. Owing to their time-varying nature, these errors cannot be directly predicted or compensated for. Instead, they are characterized by specific statistical distributions and parameters and are commonly modeled using stochastic processes [24,25].

IMU calibration methods can generally be divided into non-autonomous and autonomous approaches. Non-autonomous calibration relies on high-precision laboratory equipment, such as three-axis turntables and centrifuges, to generate known reference inputs (e.g., precise orientations, constant angular velocities, or controlled temperature gradients). By correlating the sensor outputs with these reference values, calibration algorithms can estimate intrinsic error parameters. The key advantage of this approach lies in its ability to provide accurate and repeatable reference signals. However, it typically involves high equipment costs, complex operational procedures, and limited applicability outside controlled laboratory settings.

In contrast, autonomous calibration eliminates the need for specialized equipment by utilizing environmental cues (e.g., gravity or Earth’s magnetic field) or exploiting the inherent motion characteristics of the sensor. Error parameters are estimated through mathematical modeling or data-driven algorithms. Recently, learning-based approaches, such as Calib-Net, have emerged, which directly learn the mapping from raw IMU measurements to ground-truth states without requiring explicit physical models. These methods have demonstrated strong potential in compensating for complex, nonlinear scale factor errors.

Several studies have provided comprehensive reviews of intrinsic calibration techniques for IMUs. Ru et al. [23] surveyed calibration strategies for MEMS inertial sensors, including error modeling, calibration procedures, and practical deployment in real-world applications. Poddar et al. [11] examined various IMU calibration approaches, classifying them into laboratory-based, in situ, and sensor fusion-based categories, and systematically evaluated the strengths and limitations of each method. Chen et al. [26] offered an in-depth review of deep learning-based inertial localization, highlighting its applications in pedestrian tracking, unmanned aerial vehicles (UAVs), ground vehicles, and mobile robotics. Notably, they introduced a novel three-level taxonomy—sensor level, algorithm level, and application level—to systematically organize deep learning techniques in inertial localization for the first time.

### 2.4. Temporal Offset Calibration

**Figure 4:**
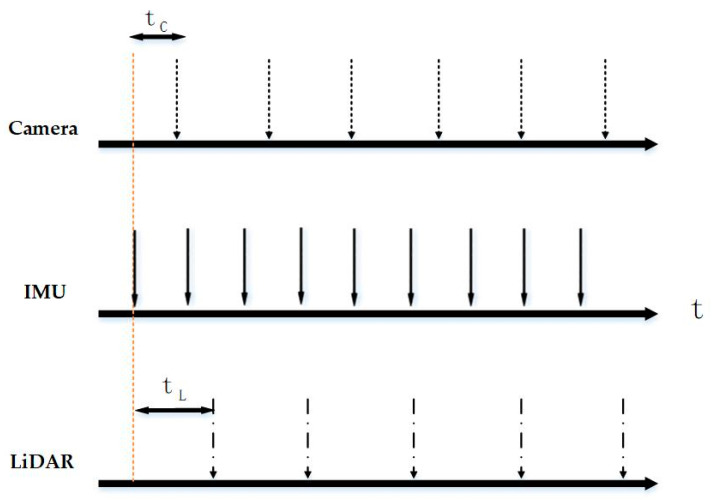
Example of time offset arising due to latency in the sensor data. t denotes the temporal flow. $t_{C}$ and $t_{L}$ represent the time delays of the camera and LiDAR relative to the IMU, respectively.

Some sensor fusion studies require highly accurate and well-aligned timestamps for camera and IMU measurements. However, these timestamps are affected by multiple factors, including differences in clock sources, triggering mechanisms, transmission delays, data congestion, jitter, and drift. Since each sensor exhibits distinct delays, temporal offsets inevitably occur, as illustrated in Figure 4. Ideally, a dedicated hardware system can synchronously trigger data acquisition for all sensors, a solution already adopted in applications demanding high-precision temporal alignment, such as the multi-sensor fusion of LiDAR, cameras, and millimeter-wave radars in autonomous driving. Nevertheless, this approach necessitates specialized hardware design.

Because temporal offsets and extrinsic parameters are independent parameters, some researchers perform time calibration separately. The most straightforward approach to estimating the temporal offset between two signals is to identify the peak of their cross-correlation [27,28]. Mair et al. [29] proposed two methods independent of spatial alignment for estimating time delays: the cross-correlation method and the phase congruency method. The cross-correlation method determines the delay by maximizing the correlation between the IMU and camera angular velocity sequences, whereas the phase congruency method estimates the delay in the frequency domain by analyzing phase congruency between signals. Experiments showed that the cross-correlation method performs reliably for small delays but yields incorrect optima for large delays. In long-delay experiments, the phase congruency method achieved a mean error of 19.3 ms, compared to 360 ms for the cross-correlation method. The accuracy of both methods remained low and requires further optimization. Qiu [30] initially estimated the temporal offset between aligned camera and IMU angular velocities using trace correlation, then refined the estimate with quadratic fitting, improving accuracy to the sub-millisecond level. Their approach achieved a mean offset error below 0.5 ms, with performance unaffected by large temporal offsets. Kelly et al. [31] treated time-delay calibration as a curve registration problem in three-dimensional orientation space, estimating the delay by aligning orientation curves generated from IMU gyroscope integration with those derived from camera observations. Similarly, Yan [32] associated the absolute rotation between the IMU and camera coordinate frames with the temporal offset, formulated a constraint equation, and applied the Gauss–Newton method to iteratively optimize the offset, minimizing inconsistencies between visual and inertial rotations.

Alternatively, the temporal offset can be modeled directly as an additional state variable, linked to sensor measurements, and jointly estimated with spatial transformation parameters. This approach supports both constant and time-varying offsets, and the uncertainty of the estimate can also be modeled. For example, Furgale [33] and Qin [34] incorporated the temporal offset into the visual reprojection error as a state variable, jointly estimating it with other states using nonlinear least squares. Liu [35] and Fu [36] added a term representing the time delay into IMU pre-integration results, and Fu [36] further derived the Jacobian coefficients of rotational, velocity, and displacement residuals with respect to the offset. Li [37] modeled the camera–IMU temporal offset as a state variable optimized within an extended Kalman filter (EKF). To address the complexity of the time-delay noise model, Peng [38] proposed an improved adaptive EKF (AEKF) based on Li’s framework, capable of online correction of noise statistics. By introducing a “forgetting factor” to balance the weights of new and historical data, this method achieved faster convergence and higher accuracy.

We summarize the experimental results of the above temporal offset calibration studies in Table 1. The comparison shows that Furgale’s [33] method achieves the smallest temporal offset error (<0.04 ms) but requires a convergence time of 300 s, making it suitable for static, high-precision applications. On the EuRoC dataset, Qin’s [34] method maintained an error between 0.01 ms and 0.3 ms across various offsets, achieving accuracy comparable to Kalibr but with faster convergence. Fu’s method produced errors of the same order of magnitude as Qin’s while yielding a lower trajectory root mean square error. Some methods demonstrated in low-offset simulations that estimation accuracy was not significantly correlated with ground truth; however, high-offset cases (e.g., >500 ms) remain unaddressed, despite their possible occurrence in real-world applications.

## 3. Extrinsic Calibration

Extrinsic calibration refers to the estimation of a rigid (Euclidean) transformation that maps three-dimensional points from one coordinate system to another, for instance, transforming points from the world or LiDAR coordinate system into the camera coordinate system. This process determines the position and orientation of a sensor relative to an external reference frame by estimating its translation and rotation along the three orthogonal axes of three-dimensional space. The resulting extrinsic parameters are typically expressed as a 3 × 4 matrix, comprising a 3 × 3 rotation matrix and a 3 × 1 translation vector.

### 3.1. Camera–IMU Extrinsic Calibration

#### 3.1.1. Decoupling-Based Methods

In early studies, the extrinsic calibration between a camera and an IMU was typically performed using specialized apparatus. Lobo and Dias [39] mounted the system on a rotation platform to estimate the direction of gravity under static conditions. Then the calibration pattern is placed on a horizontal surface and accelerometer readings are taken in the various camera poses [40]. By aligning the vertical direction in the camera coordinate system with that measured by the accelerometer in the body frame, they estimated the rotation between the IMU and the camera [41]. The system was subsequently rotated around the IMU’s center, where it experiences zero linear acceleration, which enabled estimation of the translation parameters. In practical experiments, they achieved a rotational error of 0.69° and a translational error of 5 mm using an IMU paired with a low-cost camera. This method requires precise alignment of the camera–IMU system on a dedicated rotation platform. However, accurately locating the IMU center and aligning it with the camera’s optical center is challenging. This misalignment can negatively impact overall calibration accuracy. Hol [42] adopted the approach proposed by Lobo and Dias [39] for camera–IMU calibration and sensor fusion. In his method, only the rotation parameters were estimated, while the translation parameters were derived from mechanical design specifications.

Alves [43] performed calibration of a low-cost camera–IMU system using a pendulum-based setup. The pendulum, fitted with an encoded axis, was used to estimate the bias, scale factors, and axis alignment parameters of the inertial sensor. At the same time, the camera determined the vertical direction by detecting the vanishing point of the pendulum. By combining vertical direction measurements from the IMU, through accelerometer readings, and the camera, the rotation matrix between their coordinate frames was estimated. However, this method did not account for the translation parameters.

The aforementioned camera–IMU calibration methods, relying on specialized auxiliary equipment, face significant limitations. Their dependence on such devices leads to high acquisition costs and complex system setup, while unavoidable installation and adjustment errors directly compromise calibration accuracy. Additionally, the parameter estimation process often treats rotation and translation independently, ignoring their potential coupling. This separation causes errors in rotation estimation to propagate into translation parameters, further reducing overall calibration precision. These technical challenges and practical constraints considerably limit the engineering applicability of these methods.

#### 3.1.2. Filter-Based

Filter-based calibration methods exploit filtering algorithms to fuse camera and IMU data, enabling estimation and the continuous updating of calibration parameters. Mirzaei and Roumeliotis [44] first introduced an iterative extended Kalman filter-based camera–IMU calibration approach that tracks corner points on a planar calibration target to estimate the relative poses and the bias of the IMU. However, the computational complexity of this method increases cubically with the number of environmental features, posing challenges for real-time performance in certain scenarios. Li and Mourikis [45] improved upon this by incorporating camera poses at different time steps into the state vector rather than including individual feature points, which greatly reduced the computational burden.

However, Mirzaei’s model did not account for the influence of the gravity vector. Chu and Yang [46] and Kelly and Sukhatme [47] addressed this by incorporating the gravity vector into the state estimation process. Kelly further validated the necessity of estimating the gravity vector through simulation experiments. Distinct from prior work, Kelly applied the unscented Kalman filter (UKF), which offers better performance in highly nonlinear systems. An EKF performs well when system nonlinearity is relatively mild [48]. Assuming a Gaussian distribution, the UKF can achieve third-order approximation accuracy in nonlinear state estimation. Zachariah and Jansson also applied the UKF in [49] and additionally estimated intrinsic IMU parameters, including accelerometer and gyroscope misalignments, scale factors, and bias. Later, Hartzer and Saripalli [50] implemented the EKF in a multi-camera and IMU system, and designed a two-stage filter to calibrate both temporal and spatial misalignment errors. This method effectively reduced noise and demonstrated high robustness in spatiotemporal calibration.

Within the filter-based framework, several researchers have proposed methods for calibrating shutter cameras and IMUs. Li et al. [51] proposed an error approximation method that replaces traditional motion assumptions, such as constant velocity, to address motion distortion introduced by row-wise exposure. By incorporating the intrinsic parameters of the IMU and camera, along with time delay, into the system state, they utilized an EKF to perform full online self-calibration of both the IMU and the rolling shutter camera. Lee et al. [52] employed the Cayley transformation model together with IMU measurements to compute the rolling shutter transformation. They introduced a staged gray-box Kalman filter calibration approach to estimate calibration parameters under non-fixed noise density conditions. The main idea is to estimate IMU noise characteristics using nonlinear optimization, then refine the calibration parameters based on the updated noise model. This staged framework achieves a balance between computational efficiency and estimation accuracy, leading to faster convergence compared to single-shot methods. However, the gray-box model is structurally complex and relies on limited measurement information, which makes it difficult to accurately identify and predict the underlying nonlinear system behavior. Yang et al. [53] proposed an online self-calibration method for visual-inertial navigation systems based on EKF and conducted an observability analysis, which demonstrated that a fully parameterized calibration system has four unobservable directions, corresponding to global yaw and translation. They also identified specific motion patterns, such as single-axis rotation and constant acceleration, that cause failures in calibrating the intrinsic parameters of both the IMU and the camera.

Filter-based camera–IMU calibration methods have steadily advanced, allowing for the reliable estimation of additional key parameters, including gravity, IMU intrinsic parameters, and spatiotemporal offsets. Despite these improvements, such methods depend on accurate assumptions regarding noise distribution. In certain real-time calibration scenarios, they may struggle to effectively mitigate the systematic accumulation of linearization errors. Table 2 summarizes the characteristics and contributions of selected filter-based camera–IMU calibration methods.

#### 3.1.3. Optimization-Based

Unlike filter-based methods, optimization-based approaches do not propagate the system state and covariance throughout the entire measurement sequence. Instead, they formulate a mathematical model of the sensor trajectory and obtain the optimal calibration parameters by minimizing the measurement residuals.

Dong-Si and Mourikis [54] were the first to propose an algorithm that directly computes system observability from sensor measurements. They initially estimated the rotation and translation between the camera and the IMU using a convex optimization method, and subsequently refined the results by solving a nonlinear least-squares problem with the Levenberg–Marquardt algorithm. Although this approach proved effective, it did not dynamically estimate the bias of the IMU’s gyroscope and accelerometer. As a result, accumulated errors over time degraded the calibration accuracy. Building on this work, Yang and Shen [55] introduced a sliding window framework to constrain the temporal accumulation of IMU errors during optimization. However, similar to the previous method, it did not account for IMU bias.

For estimating IMU bias, Mur-Artal and Tardos [56] proposed a stepwise method to separately estimate the bias of the accelerometer and gyroscope. Their approach achieved high accuracy within a short time frame but required prior knowledge of the extrinsic parameters between the camera and IMU. Qiu et al. [57] introduced a high-precision calibration method for camera–IMU systems based on adaptive constraints derived from multiple error equations. In their framework, IMU errors are treated as adaptive constraints embedded into error compensation equations, and the Newton method is employed to iteratively solve for the optimal model parameters. By comprehensively accounting for IMU bias and lens distortion, this method supports accurate online calibration.

Qin et al. [58] proposed a general monocular visual-inertial state estimation system, VINS-Mono. This system employs structure from motion (SfM) and IMU pre-integration to perform online estimation of gyroscope bias, gravity vector, scale parameter, and camera–IMU extrinsic parameters. It overcomes the limitation of traditional VINS, which necessitates static initialization or slow movement. Similarly, Huang and Liu [59] proposed an online calibration method that does not rely on any prior information. They employed a three-stage strategy to progressively optimize the calibration parameters and to automatically identify their convergence.

However, the aforementioned methods did not account for the temporal offset between camera and IMU data. Fleps et al. [60] were among the first to model camera–IMU extrinsic calibration as a nonlinear batch optimization problem by using B-spline curves to parameterize the IMU trajectory. They incorporated time delay into the measurement model and solved the problem using a sequential quadratic programming (SQP) algorithm. Due to the batch-processing nature of this method, it is inherently limited in real-time applications. Furgale et al. [33] later introduced the well-known Kalibr toolbox, which formulates a continuous-time joint calibration model based on maximum likelihood estimation. This method employs B-splines to parameterize the time-varying IMU states and directly embeds the temporal offset as an optimization variable within the measurement model. Thus, it avoids the estimation difficulties caused by timestamp misalignments in discrete-time models. Finally, a least-squares optimization framework was constructed by integrating image, IMU measurement residuals, and bias to solve for the spatiotemporal parameters of the camera–IMU. Building on this work, Li and Mourikis [37] demonstrated that the camera–IMU time offset can be effectively estimated by modeling it as an additional state variable, thus providing a theoretical basis for addressing sensor time asynchrony issues. Rehder et al. [61] extended the Kalibr framework to multi-IMU systems, and Huai et al. [62] adapted it for use with rolling-shutter cameras. More recently, Nikolic et al. [63] proposed a non-parametric joint calibration approach that differs from Kalibr by eliminating the need to predefine temporal basis functions or motion noise parameters. Their method incorporates the discrepancy between continuous IMU integration and discrete sampling into the maximum likelihood framework, enabling joint estimation of trajectory and calibration parameters without relying on strict prior assumptions.

Optimization-based camera–IMU calibration methods directly solve nonlinear cost functions using nonlinear optimization algorithms such as Levenberg–Marquardt or Gauss–Newton, thereby avoiding the inherent errors introduced by linearization in filtering-based approaches. These methods can simultaneously process a large amount of measurement data, resulting in higher accuracy and robustness. However, their computational cost is relatively high, making them less suitable for real-time applications compared to filter-based methods. Table 3 summarizes the characteristics and contributions of selected optimization-based camera–IMU calibration methods.

#### 3.1.4. Learning-Based

Traditional calibration approaches typically involve carefully designed pipelines, encompassing feature extraction, matching, and parameter optimization, all of which rely heavily on manually engineered rules and algorithms. Deep learning provides a promising alternative for camera–IMU calibration by leveraging large-scale data to learn complex feature representations and mapping relationships. Deep learning-based extrinsic calibration methods can be broadly categorized into two main strategies. The first involves substituting individual modules within the conventional pipeline, such as feature extraction or matching. The second seeks to model and optimize the nonlinear parameters of the sensor to improve the calibration accuracy.

Guo et al. [64] retained the traditional feature extraction and matching process in their calibration framework. They used the rotation matrices of adjacent images along with 10 sequences of IMU measurements as input to a backpropagation neural network. The network was trained by minimizing the mean squared error using the Levenberg–Marquardt algorithm, enabling direct mapping from IMU data to camera pose. Some work focuses on enhancing calibration accuracy by refining IMU intrinsic parameters. For instance, Liu et al. [65] proposed a dual-branch dilated convolutional network that directly estimates the scale factors of the gyroscope and accelerometer, as well as accelerometer correction terms, including bias and noise. However, their approach depends on ground-truth position and orientation data as supervision. To address the challenge of limited supervision, Hosseini et al. [66] employed a loosely coupled error-state extended Kalman filter (ESKF) [67] to fuse IMU and camera data, thereby generating reliable training targets. A convolutional neural network (CNN) was then trained as a front-end calibration module to produce bias and noise-compensated IMU data, which was subsequently refined in real-time by ESKF.

In recent years, deep learning-based research on IMU intrinsic calibration has achieved notable advancements. Brossard et al. [68] proposed the use of deep neural networks (DNNs) to model IMU noise characteristics and to dynamically adjust filter parameters, thereby enhancing estimation accuracy. Zhang et al. [69] employed DNNs to compute observable IMU integration terms, improving both robustness and precision. Gao et al. [70] proposed Gyro-Net to estimate and compensate for random noise in gyroscope measurements. Buchanan et al. [71] conducted a comparative study of long short-term memory (LSTM) and transformer-based architectures for IMU bias compensation. A lightweight convolutional neural network, Calib-Net, was developed by Li et al. [72], utilizing dilated convolutions to extract spatiotemporal features from IMU measurements and output dynamic compensation terms for gyroscope readings. This framework enables high-precision calibration for low-cost IMUs.

In the area of end-to-end visual-inertial odometry (VIO), Clark et al. [73] introduced VINet, a framework that directly estimates sensor poses by processing raw visual and inertial data through a neural network, without requiring manual extrinsic calibration or clock synchronization. However, this system depends on ground-truth poses from high-precision reference platforms, which limits its practical deployment. To overcome the challenges of scale ambiguity in monocular vision and the scarcity of supervised ground-truth data, Han et al. [74] used stereo image pairs to generate three-dimensional geometric constraints that provide absolute scale supervision. They developed DeepVIO, a self-supervised deep learning-based VIO system that operates using only monocular images and IMU data, thereby eliminating the need for extrinsic calibration.

Some notable studies have omitted the extrinsic calibration step. In end-to-end visual-inertial odometry, Clark et al. [73] proposed VINet, a neural network that takes raw images and IMU data as input and directly outputs the pose, thereby removing the need for manual extrinsic calibration and clock synchronization. However, this approach depends on high-precision systems to provide ground-truth poses, which limits its practical applicability. Han et al. [74] developed DeepVIO, a self-supervised, deep learning-based visual-inertial odometry method that also operates directly on monocular images and IMU data without requiring extrinsic calibration. This method has demonstrated robustness under challenging conditions, including inaccurate camera–IMU calibration, unsynchronized data, and data loss. Learning-based methods show considerable potential; however, several unresolved issues, including reliance on labeled data, limited global consistency, and poor interpretability, hinder their ability to replace traditional approaches in the near term.

#### 3.1.5. Discussion

The four categories of camera–IMU calibration methods exhibit distinct trade-offs in terms of accuracy, computational efficiency, and adaptability to various environments. Methods based on decoupled models reduce computational complexity by separating the calibration of rotation and translation; however, their dependence on specialized equipment and disregard for coupling effects may lead to significant accuracy degradation. Filter-based methods employ recursive estimation techniques that support both online and offline calibration and demonstrate strong adaptability to dynamic environments. Nevertheless, inaccurate assumptions about the noise model can cause the estimation to diverge. Optimization-based methods jointly solve visual-inertial constraints to achieve high calibration accuracy, although they are often computationally intensive. Deep learning methods automate calibration by leveraging large-scale datasets, but their performance is highly dependent on labeled data and tends to degrade when applied to previously unseen scenarios. Table 4 summarizes the principles, advantages, and limitations of each approach, along with relevant references.

To further evaluate camera–IMU calibration performance, we compared the calibration performances of several camera–IMU methods in Table 5. Studies that did not explicitly report camera–IMU extrinsic parameter errors were excluded, as their experiments may not have been specifically designed for camera–IMU extrinsic calibration or may have validated their methods through other types of experiments. In general, methods with smaller calibration errors are considered superior, although exceptions occur. For example, Lee’s [52] method yielded smaller calibration errors than that of Qiu [57], yet Qiu [57] experimentally demonstrated superior overall performance. This discrepancy arises from the lack of standardization in datasets and hardware across studies. Factors such as the level of motion excitation during data collection, the density of environmental features, camera resolution, IMU accuracy, and even the number of algorithm iterations can all influence calibration outcomes. Optimization-based methods, exemplified by Kalibr [33], typically achieve high precision, with the best results reporting an average translation error of less than 1 mm and an average rotation error of less than 0.01°, although this comes at the cost of real-time performance. The trade-off between accuracy and computational efficiency remains an important topic for future investigation.

Following the evaluation approach in [75], we also assessed the robustness of these algorithms from four perspectives:

(A) Whether experiments tested the method on multiple dataset types or real-time data. Methods tested on two or more types received two stars (✩✩).

(B) Whether tests included degenerate or challenging scenarios. If so, one star was awarded (☆).

(C) Whether the range of initial errors in the test data was sufficiently large. If the initial average translation exceeded 50 mm or the initial average rotation exceeded 10°, one star was awarded (☆).

(D) Whether anti-noise testing was conducted. If so, one star was awarded (☆).

Under this scheme, the maximum possible score was five stars. Some methods achieved four stars, indicating strong robustness.

Recent advancements in camera–IMU calibration have shifted research focus toward multi-sensor systems and task-specific adaptations. In the field of multi-camera and IMU calibration, Eckenhoff et al. [76] proposed an online approach based on the MSCKF framework, which aligns asynchronous timestamps and jointly optimizes the geometric constraints of multiple cameras. This method eliminates the dependence on overlapping FoVs and improves robustness in dynamic environments. Bo Fu et al. [77] developed the Mu-CI system, which minimizes a multi-objective cost function comprising inertial residuals, reprojection errors, and AprilTag association errors.

Calibration methods for specific tasks are often tailored to the operational environment. To address the instability of checkerboard corner detection in underwater welding scenarios, Chi [78] proposed a prediction–detection corner algorithm and employed joint optimization of intrinsic and extrinsic parameters to enhance underwater calibration accuracy. In unmanned aerial vehicle (UAV) applications, Yang et al. [79] analyzed the observability conditions of camera boresight misalignment angles using the observability Gramian matrix and designed an online trajectory optimization algorithm with physical constraints by optimizing the relative geometry between the UAV and the target. For autonomous driving vehicles, Xiao et al. [80] proposed an extrinsic calibration quality monitoring algorithm based on online residual analysis. In multi-sensor fusion calibration, researchers have extended calibration techniques to include additional modalities such as LiDAR, GNSS [81,82,83], wheel encoders [84], and ultra-wideband (UWB) systems [85]. Calibration strategies involving LiDAR will be discussed in Section 3.4.

### 3.2. LiDAR–IMU Extrinsic Calibration

To calibrate the extrinsic parameters between a rigidly connected LiDAR and IMU, a commonly adopted strategy is to align their respective trajectories. Geiger et al. [86] proposed a motion-based method that estimates the transformation through hand–eye calibration [87]. Schneider et al. [88] utilized an unscented Kalman filter to compute the transformation between two odometry-based sensors. Xia et al. [89] estimated the extrinsic parameters by minimizing the Euclidean distance between LiDAR point clouds transformed using IMU data and those aligned through the iterative closest point (ICP) algorithm. Despite their effectiveness, these approaches often overlook the accumulated error and rotational drift introduced by the IMU under real-world conditions.

Gentil et al. [90] modeled LiDAR–IMU calibration as a factor graph optimization problem. They used Gaussian process regression [91] to upsample IMU measurements and correct motion distortion in LiDAR scans. The upsampled IMU data were pre-integrated, and the system jointly minimized point-to-plane residuals and IMU errors to estimate the extrinsics. Li et al. [92] refined this approach by introducing an improved time offset model in the pre-integration process, computing initial rotation through a closed-form solution and using dynamic IMU initialization to estimate gravity and velocity. However, this method requires a dense prior point cloud map to extract planar features, which may limit its applicability in real-world scenarios. Zhu et al. [93] proposed a real-time initialization framework, LI-Init, which first estimates a coarse temporal offset by maximizing the correlation of angular velocity magnitudes between LiDAR and IMU. It then aligns LiDAR odometry with IMU measurements for further refinement. The method also evaluates motion excitation by analyzing the singular values of the Jacobian matrix. Yang et al. [94] further improved calibration accuracy by applying a point-based update strategy [95] to correct distortion in the LiDAR scan during motion.

Mishra et al. [96] observed that, although [90] modeled IMU data using Gaussian processes, the factor graph performed optimization only at discrete time steps, which may reduce accuracy. To address this issue, they proposed an EKF-based calibration method integrated into the OpenVINS [97] visual-inertial framework. This method does not rely on calibration targets or structured environments. However, the EKF is sensitive to the initial state, and inaccurate initial rotation estimates can propagate through the filter and degrade final calibration performance.

Continuous-time batch optimization, based on temporal basis functions, has also been widely adopted. Furgale and Rehder conducted a series of foundational studies in spatiotemporal calibration. In [61], they implemented a complete SLAM system using B-spline basis functions for camera–IMU calibration. This framework was later extended to support both temporal and spatial calibration [98], and further generalized to camera–LiDAR–IMU systems by integrating point-to-plane constraints [99] into the batch optimization. A key limitation of this approach is its reliance on highly accurate visual-inertial trajectories, which restricts its applicability to LiDAR–IMU calibration. LV et al. [100] modeled IMU states as continuous-time splines, enabling accurate pose estimation at each LiDAR scan time. Unlike [90], their method exploited all planar surfaces in the environment by projecting LiDAR points onto corresponding planes and incorporating these projections as constraints in a nonlinear least-squares optimization. Although this approach does not require artificial targets, it depends on environments rich in planar features. Their later work, OA-Calib [101], improved computational efficiency by selecting high-information trajectory segments and introduced an observability-aware update mechanism to ensure robustness under degenerate motion. Wu et al. [102] extended this by dynamically adjusting frame length in LiDAR odometry to maintain near-linear motion and scene stability, thereby reducing nonlinearity and environmental variation.

Some recent methods are based on dedicated equipment. Liu et al. [103] designed a calibration system using cone cylinder structures. They estimated point cloud poses relative to these shapes and formulated an optimization problem with geometric constraints to solve for extrinsics. Subsequently, they proposed using features such as points, lines, spheres, cylinders, and planes extracted from LiDAR scans in natural scenes [104]. While this method avoids specially designed targets, its effectiveness is limited in scenes where such features are scarce or unreliable.

Online LiDAR–IMU calibration has also been integrated into SLAM systems. [105,106] fused LiDAR, IMU, and camera data using lightweight EKF frameworks, where extrinsic parameters are estimated online. Ye et al. [107] proposed a tightly coupled odometry system that jointly minimizes residuals from LiDAR points and IMU pre-integration for real-time extrinsic estimation. Xu et al. [108] introduced FAST-LIO2, which performs iterative EKF-based calibration during tightly coupled LiDAR–IMU odometry. These online methods typically assume reasonable initial estimates for convergence. Since extrinsic parameters are included in the state vector [105,109], unobservable directions may emerge under certain motions. To address this, Kim et al. [110] proposed GRIL-Calib, a method tailored for ground robots with planar motion. It introduced geometric constraints, such as enforcing the orthogonality between the ground normal vector in the LiDAR frame and the ground *z*-axis, to constrain roll and pitch. It also assumed a constant vertical offset between the IMU and LiDAR to constrain z-translation.

Similarly, we compared the calibration performance of several LiDAR–IMU methods in Table 6. By contrasting these results with those from camera–IMU calibration, it is evident that LiDAR–IMU calibration generally achieves slightly lower accuracy. This difference arises because image features, such as corner points, are highly dense, providing over-constrained conditions for camera pose estimation and enabling highly precise optimization. In contrast, point cloud features, such as planes and edges, are relatively sparse, with only a few dozen valid geometric features per frame. Consequently, the corresponding registration error models lack sufficient constraints, making them more susceptible to local minima. For instance, Xia [89] adopted a trajectory alignment approach, in which the algorithm relies on the iterative closest point (ICP) method to establish point cloud correspondences. When initial errors are large, the algorithm can converge to local minima, resulting in absolute translation and rotation errors of 17.2 mm and 1.2°, respectively.

Another noteworthy approach is GRIL-Calib [110], proposed by KIM et al., whose calibration results are not directly comparable to those of other methods. GRIL-Calib was evaluated in a ground-robot planar-motion scenario, where the absence of excitation along the vertical (*z*-axis) inherently renders certain parameters, such as roll and pitch rotations, as well as the *z*-axis translation, unobservable. In contrast, other methods were tested under full-axis motion excitation, which provides superior parameter observability. To address this unobservability in planar motion, GRIL-Calib introduces the ground planar motion (GPM) constraint. However, this method relies heavily on the geometric regularity of a flat ground surface. In non-planar environments, such as slopes or steps, reduced accuracy in ground segmentation can cause the geometric relationships defined by the GPM constraint to break down, thereby degrading calibration performance.

Based on experimental results reported in other studies, we have also summarized the following three important conclusions:

- Regardless of whether LiDAR operates in 128-, 64-, 32-, or 16-channel mode, the algorithm converges to comparable calibration parameter estimates, indicating insensitivity to LiDAR point cloud density [96].
- Experimental comparisons show that, without point cloud de-skewing, calibration results fail to converge and exhibit large fluctuations. When de-skewing is performed using IMU state prediction, scan matching accuracy improves significantly, and calibration parameters converge stably [96].
- During data collection, all rotational and translational degrees of freedom of the sensor suite must be sufficiently excited to ensure full observability of the extrinsic calibration parameters. Experiments demonstrate that, with adequate motion excitation, both translation and rotation parameters converge rapidly; insufficient excitation may result in biased estimates [96,110].

LiDAR–IMU calibration technology was previously overlooked due to the high computational complexity involved in processing LiDAR point clouds. However, with the increasing demand for LiDAR–IMU fusion in recent years, research in this area has grown substantially. Nevertheless, the calibration accuracy of LiDAR–IMU systems remains lower than that of camera–IMU systems, leaving room for further improvement. Table 7 presents several representative calibration approaches, along with their main contributions.

### 3.3. Camera–LiDAR Extrinsic Calibration

The calibration between LiDAR and cameras has been extensively reviewed by numerous scholars in recent years. Readers seeking a more comprehensive and in-depth understanding of camera–LiDAR calibration are encouraged to consult the survey papers listed in Table 8. Liu et al. [111] were the first to systematically summarize the development of camera–LiDAR calibration, outlining the complete calibration process and describing the principles and algorithms for estimating both intrinsic and extrinsic parameters. Li et al. [112] reviewed the progress of targetless calibration methods and categorized existing approaches into four main groups: information-theoretic methods, feature-based methods, ego motion-based methods, and learning-based methods. This study analyzed the theoretical foundations, advantages, limitations, and application scenarios of each category. Qiu et al. [113] provided an overview of extrinsic calibration methods for imaging sensors, including cameras, LiDAR, and millimeter-wave radar. Liao et al. [8] conducted a systematic review of the application of deep learning in camera calibration, which includes LiDAR–camera calibration. Tan et al. [75] focused specifically on deep learning-based calibration methods, classifying them into accurate extrinsic parameter estimation (AEPE) and relative extrinsic parameter prediction (REPP), and established a structured knowledge system in this area. Zhang et al. [114] offered a detailed summary of 2D–3D, 3D–3D, and 2D–2D matching techniques for camera–LiDAR calibration, covering sensor modeling, error analysis, and key methodologies. The review included both traditional and deep learning-based approaches. Based on these survey studies, we categorize existing camera–LiDAR calibration techniques into four types, as summarized in Table 9, and compare their respective advantages and disadvantages.

### 3.4. Camera–LiDAR–IMU Extrinsic Calibration

**Figure 5:**
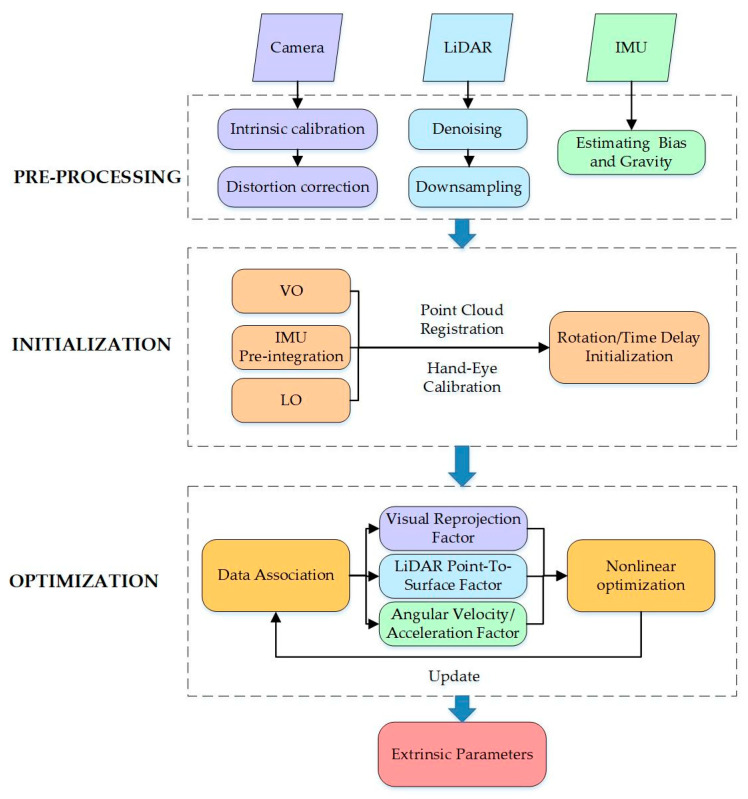
General calibration procedure for camera, LiDAR, and IMU.

Traditional calibration of camera–LiDAR–IMU (LVI) systems typically follows a pairwise, chain-based strategy in which the extrinsic parameters are estimated sequentially between sensor pairs to derive the complete spatial and temporal relationships among the three sensors. However, this step-by-step approach introduces operational redundancy and allows errors to accumulate at each stage, since every sensor pair requires a customized calibration process. To address these limitations, joint calibration methods have been developed to estimate the spatiotemporal parameters of all three sensors within a unified framework. The general procedure of such methods can be divided into three stages: preprocessing, parameter initialization, and nonlinear optimization, as illustrated in Figure 5. After the data from each individual sensor is preprocessed, initial estimates of rotation and temporal offset are obtained through pose estimation, point cloud alignment, and hand–eye calibration. Finally, multiple cross-modal constraints, including visual reprojection errors, LiDAR point-to-plane correspondences, and IMU kinematic residuals, are jointly optimized using iterative nonlinear techniques to accurately estimate the extrinsic parameters.

Several researchers have introduced target-based calibration approaches. Hou et al. [125] extracted line and plane features from a checkerboard to establish geometric constraints between the camera and LiDAR. These constraints were then translated into constraints on the camera–IMU and LiDAR–IMU extrinsics, which were jointly optimized with IMU measurement residuals, enabling simultaneous estimation of both sets of extrinsic parameters. Zhi et al. [126] introduced a method compatible with multiple cameras, LiDAR sensors, and IMUs, even in scenarios without overlapping FoVs. Their approach utilizes multiple planar calibration targets with AprilTags. By incorporating LiDAR point-to-plane constraints, IMU measurements, and corner reprojection errors, the system jointly estimates all extrinsic parameters without relying on any prior spatial initialization.

More recently, targetless spatiotemporal calibration methods based on continuous-time frameworks have gained attention. Liu et al. [127] employed an IMU-centric approach, using correlation analysis and hand–eye calibration to obtain the initial estimates of the time offset and extrinsic rotation. They then calibrated the extrinsic parameters between the IMU and LiDAR, optimized the IMU trajectory, and subsequently calibrated the extrinsic parameters between the IMU and camera.

Wang et al. [128] integrated multiple constraints into a unified optimization framework, including point-to-surfel constraints, visual reprojection errors, a visual point-to- LiDAR-surfel constraint, and the error constraint between IMU measurements and trajectory derivatives. However, this requires an accurate initial estimate of the camera–IMU extrinsic parameters to construct the visual-inertial odometry (VIO) system. In addition, due to its computational complexity and scale, each calibration takes approximately 200 s, which limits its suitability for real-time applications. However, in [129], the LiDAR point-to-plane, gyroscope, and accelerometer factors are jointly optimized to estimate the LiDAR–IMU extrinsic parameters, time offset, and the poses of control points. Then, by incorporating the visual reprojection factor, the control points in the spline trajectory are fixed to calibrate the camera–IMU extrinsic parameters and time offset. Li and Chen et al. [130] combined LiDAR point-to-plane and visual reprojection factors into a single optimization process to solve for both sets of extrinsic parameters simultaneously. Their approach also supports rolling shutter cameras. Wang et al. [131] proposed a parallel calibration method for LiDAR–IMU and camera–IMU extrinsics, including convergence criteria and excitation evaluation metrics. They developed a user-friendly, targetless online calibration framework for camera–LiDAR–IMU systems, demonstrating notable improvements in both accuracy and efficiency compared to existing methods.

In contrast, Wang and Ma [132] proposed a non-continuous-time approach with significantly improved efficiency. In the first stage, they estimated the camera–IMU extrinsics, time offset, and IMU bias to obtain reliable VIO results. In the second stage, the VIO trajectory was aligned with LiDAR odometry obtained using the iterative closest point (ICP) method to calibrate the LiDAR–IMU extrinsics. Their method, evaluated on the EuRoC dataset, achieved superior performance compared to VINS-Mono. The total calibration time, including both VIO and LiDAR–IMU initialization, ranged from 10 to 20 s, although the accuracy was slightly lower than that of offline methods.

Despite advances in multi-sensor fusion technologies, research on the joint calibration of LiDAR, cameras, and IMU systems remains limited. Table 10 summarizes the characteristics and contributions of representative LVI calibration methods. Table 11 presents the calibration performance of several camera–LiDAR–IMU methods. Compared with the dual-sensor calibration results (camera–IMU and LiDAR–IMU) presented in the other two tables, the accuracy of three-sensor configurations is generally comparable, but their convergence time is markedly longer. Robustness evaluations further reveal that current three-sensor calibration methods have not been adequately validated in challenging environments, such as low-texture scenes, dynamic occlusions, or adverse lighting conditions. Nevertheless, by integrating the visual detail of cameras, the three-dimensional structural perception of LiDAR, and the motion continuity of IMUs, three-sensor systems inherently provide a more comprehensive environmental perception capability, theoretically offering a natural adaptability advantage for calibration in complex scenarios. Future research directions include developing calibration methods tailored to specific challenging conditions (e.g., tunnels, rainy nights, or dynamic crowd interference), advancing online real-time calibration frameworks and addressing generalized calibration across heterogeneous devices (e.g., combinations of consumer-grade and industrial-grade sensors) to fully exploit the potential of multimodal fusion.

## 4. Discussion

### 4.1. Key Evaluation Metrics of Calibration Methods

In the evaluation of multimodal sensor calibration methods, accuracy alone is not the sole criterion. A balanced consideration of computational efficiency, level of automation, and robustness is essential in determining the practical value of a calibration approach.

#### 4.1.1. Automaticity

A key characteristic of automatic calibration is its ability to operate without human intervention. Target-based calibration employs predefined geometric information to achieve efficient and accurate cross-sensor data association. Common examples include visual markers such as checkerboards [40] and AprilTags [12] for cameras, as well as planar targets [133] or corner reflectors that are easily detected by LiDAR. These methods typically attain sub-pixel or centimeter-level accuracy due to the presence of strong prior constraints. However, this comes at the cost of convenience.

Targetless calibration automatically extracts natural features from the environment to establish cross-modal constraints. Representative examples include wall edges in indoor environments, road boundaries on highways, and building facades in urban areas. Such methods are sensitive to environmental conditions. In textureless or open areas, feature extraction may fail, leading to calibration errors. Moreover, noise in natural features, including interference from dynamic objects, can introduce cumulative inaccuracies.

#### 4.1.2. Efficiency

Most existing calibration algorithms depend on iterative optimization to achieve high accuracy, which often results in high computational cost and long processing time. To handle highly dynamic scenarios, some methods adopt continuous-time models to fit the IMU trajectory, further increasing the computational burden. In online calibration, the estimation of spatiotemporal parameters is coupled with real-time state estimation tasks such as SLAM and odometry. If the calibration process consumes excessive system resources, it may adversely affect the stability of these concurrent tasks. While computational inefficiency is less critical in offline settings, extended calibration durations remain a significant limitation. The convergence times of some algorithms have already been presented in Table 5, Table 6 and Table 11, which summarize the calibration performance.

#### 4.1.3. Robustness

Sensitivity to initial values, noise resilience, and environmental adaptability are critical indicators for evaluating the robustness of calibration algorithms, particularly in targetless approaches. Target-based methods are excluded from this evaluation because they rely on reference objects and typically do not require an initial value. In targetless calibration, the accuracy of feature extraction and matching directly influences calibration performance. This process is highly susceptible to environmental factors, including the number of features, lighting conditions, weather, and the presence of dynamic objects. To date, no calibration methods have been extensively tested in highly challenging environments, although some studies have evaluated performance under degraded conditions [101,110].

Additionally, sensitivity to initial values remains a major issue. A reasonable initial estimate can help the algorithm avoid local minima and speed up convergence. In practical scenarios, initial values may be inaccurate and measurements noisy, necessitating algorithms with strong self-calibration capabilities that can handle parameter initialization without prior information. Only a few methods have been evaluated under substantial initial errors, specifically with average initial translation exceeding 50 mm or average initial rotation exceeding 10° [48,61,62].

We present the robustness evaluation of these algorithms in Table 5, Table 6 and Table 11 (the evaluation methodology is detailed in Section 3.1.5). No algorithm achieved a full five-star rating. Only a few methods that combined simulated and real-world experiments, noise-resilience testing, and sufficiently large initial error ranges received four stars. Although this rating system cannot fully capture an algorithm’s robustness, it highlights that current research on algorithmic robustness remains limited.

### 4.2. Open-Source

With the rapid advancement of multi-sensor calibration techniques, numerous high-performance algorithms have been developed. Corresponding open-source datasets have also emerged continuously. To facilitate further progress in robotics and autonomous driving, many researchers have released both their methods [134,135,136,137,138,139,140,141,142,143,144,145,146,147,148] and the associated datasets, improving accessibility and supporting ongoing innovation. However, these open-source implementations and datasets for different sensor combinations are often dispersed across various publications, and some lack clear categorization. This fragmentation makes it difficult for researchers to efficiently identify and select implementations that best suit their specific needs. To address this problem, we compile a collection of open-source implementations and open datasets referenced in calibration-related studies, as summarized in Table 12 and Table 13. The table includes key information for each project, such as the corresponding citation, supported sensors, whether the method is target-based, and other relevant details, with dataset entries also providing download links. This compilation provides a convenient reference for accessing suitable tools and aims to accelerate research in multi-sensor fusion.

### 4.3. Future Research

#### 4.3.1. Low-Cost Devices Adaptation

Most existing studies focus on sensors with high observation accuracy, where precise calibration can be achieved under simplified noise models. In contrast, calibrating low-cost consumer-grade sensors faces significant challenges due to inherent performance limitations. For example, low-cost IMUs often suffer from considerable bias drift and scale factor noise [174]; low-cost LiDAR typically produces sparse point clouds that lack adequate structural features [136]; and the associated cameras are usually characterized by low resolution and severe lens distortion. These non-ideal properties violate the assumptions of stationary noise that conventional calibration methods depend on, thereby degrading the overall calibration accuracy.

#### 4.3.2. Environment

Research on targetless calibration methods has mainly concentrated on environments with abundant point, line, and plane features, such as indoor areas, highways, and urban settings. However, in degraded environments characterized by low texture, weak structural elements, dynamic occlusions, or elevated sensor noise, as well as in adverse conditions, including extreme lighting, rainfall, snowfall, or complex terrain, the performance of existing algorithms often declines due to unstable feature extraction, limited geometric constraints, and unsuccessful cross-modal data association [175]. In practical applications, sensor systems are frequently deployed in unstructured outdoor environments and dynamic, all-weather conditions [107]. These situations involve uncertain environmental features and complex interference, which poses significant challenges for multi-sensor calibration.

#### 4.3.3. Online Calibration

Offline calibration requires prior data collection and post-processing, making it difficult to adapt to scenarios where sensor parameters vary dynamically over time or under changing environmental conditions. In automotive applications, for instance, vibrations can alter sensor mounting poses, and temperature fluctuations may cause lens distortion drift, both of which can quickly render offline calibration results invalid [109]. In contrast, online calibration employs real-time, data-driven parameter updates to continuously compensate for spatiotemporal misalignments between sensors. This enables accurate alignment of multimodal data in time-critical tasks such as SLAM and object tracking. At present, online calibration techniques for dual-sensor configurations, such as camera, IMU, and LiDAR–IMU, are relatively mature [51,53,93]. However, joint online calibration involving all three sensors (camera, LiDAR, and IMU) still faces significant technical challenges. These include robust data association across modalities and constraints imposed by limited computational resources in real-time systems.

#### 4.3.4. Deep Learning

Deep learning is undoubtedly one of the most prominent research directions today. Traditional methods extract handcrafted features such as points, lines, and planes, but they suffer from significant limitations in environmental understanding. These features tend to be unstable in unstructured or varying-resolution scenarios, and their capacity to represent environmental semantics is limited, making it difficult to support deep cross-modal data association and comprehensive environmental perception.

## 5. Conclusions

In conclusion, this paper presents a comprehensive review of multimodal sensor calibration techniques involving cameras, Light Detection and Ranging (LiDAR), and inertial measurement units (IMUs). As a critical prerequisite for multi-sensor fusion, accurate spatiotemporal calibration among sensors is essential. This review systematically summarizes recent advancements in extrinsic calibration methods, encompassing camera–IMU, LiDAR–IMU, camera–LiDAR, and integrated camera–LiDAR–IMU systems. Among these, camera–IMU calibration has reached a relatively mature stage, with current research efforts shifting toward extending system functionality and meeting task-specific demands. In contrast, both LiDAR–IMU and camera–LiDAR calibration continue to face challenges such as sparse environmental structures and interference from dynamic scenes. The joint calibration of camera–LiDAR–IMU systems introduces additional complexity due to the high-dimensional state space and tightly coupled parameters, yet it holds significant potential for future research. In addition, this paper discusses key evaluation metrics for calibration methods and compiles a set of open-source implementations and datasets for camera–LiDAR–IMU calibration, aiming to reduce redundant development efforts. Based on the analysis of these metrics and tools, it is clear that no single calibration method can meet the needs of all application scenarios. Therefore, calibration strategies should be selected according to specific use cases. This review serves as a technical reference for researchers and engineers working on multimodal sensor systems and aims to support further progress while outlining future research directions.

## Figures and Tables

**Table 1:** Results of temporal offset calibration.

Method	Year	Convergence Time (s)	Datasets	Ground Truth of Time Offset (ms)	Temporal Offset Error (ms)	RMSE ^1^ (m)
Mair [29]	2011	-	Self-Record	500	[19.3, 360]	-
Qiu [30]	2021	10	EuRoc:V1_01	0	[0.029, 0.633]	-
Kelly [31]	2014	5–8	Self-Record	25	[0.93, 2.12]	
Qin [34]	2018	<5	EuRoc	5	[0.01, 0.16]	[0.155, 0.303]
				15	[0.02, 0.3]	[0.158, 0.326]
				30	[0.01, 0.21]	[0.156, 0.303]
Liu [35]	2018	-	EuRoc	−30	<0.25	[0.338, 0.654]
				0	<0.25	[0.336, 0.656]
				30	<0.25	[0.338, 0.653]
				60	<1	[0.336, 0.657]
Fu [36]	2022	10	EuRoc	−30	[0.09, 0.48]	[0.019, 0.076]
				−15	[0.03, 0.47]	[0.022, 0.062]
				15	[0.07, 0.27]	[0.016, 0.069]
				30	[0.07, 0.76]	[0.020, 0.079]
Peng [38]	2023	10	EuRoc:MH05	10	-	0.666
Li [37]	2014	-	Self-Record	0	[0.1, 1.519]	-
Furgale [33]	2013	300	Self-Record	-	<0.04	-

^1^ RMSE is root mean square error.

**Table 2:** Characteristics and Contributions of Selected Filter-Based Camera–IMU Calibration Methods.

References	Estimator ^1^	Target ^2^	Contributions	Open-Source	Year
Mirzaei [44]	Online	Based	EKF-based; proved the system’s observability using Lie derivatives		2008
Li [45]	Online	Free	Proposed incorporating camera poses at different time steps into the state vector for joint optimization		2013
Kelly [47]	Offline	Free	The first to achieve simultaneous joint estimation of the 6-DOF camera–IMU transform, IMU bias, gravity vector, and the metric scene structure		2011
Hartzer [50]	Online	Based	Extended camera–IMU calibration to multiple cameras, enabling calibration in scenarios with non-overlapping FoV of multiple cameras	✓	2022
Li [51]	Online	Free	Proposed an error approximation approach for calibrating rolling shutter cameras and IMUs; incorporated camera intrinsic parameters into the calibration framework		2014
Lee [52]	Offline	Based	Proposed methods under non-fixed noise density conditions		2018
Yang [53]	Online	Free	Online full-parameter calibration of a rolling shutter camera and an IMU		2022

^1^ Estimator: Whether the calibration is online or offline. ^2^ Target: Whether artificial targets are required.

**Table 3:** Characteristics and contributions of some optimization-based camera IMU calibration methods.

References	Optimization ^1^	Estimator ^2^	Target ^3^	Contributions	Open-Source	Year
Dong Si [54]	Discrete	Offline	Free	Proposed an initialization method for rotation parameters that is adaptable to different numbers of feature points		2012
Mur-Artal [56]	Discrete	Online	Free	Developed a stepwise initialization method for IMU bias		2017
Qiu [57]	Discrete	Offline	Based	Used adaptive constraints derived from multiple error equations		2020
VINS-Mono [58]	Discrete	Online	Free	Online calibration of gyroscope bias, estimation of the gravity vector, scale factor, and camera–IMU extrinsic parameters	✓	2018
Fleps [60]	Continuous	Offline	Based	Formulated the camera–IMU extrinsic calibration problem as a nonlinear batch optimization task; parameterized the IMU trajectory using B-spline curves		2011
Kalibr [33]	Continuous	Offline	Based	Proposed a continuous-time batch estimation framework; integrated temporal and spatial transformation into a maximum likelihood estimation framework	✓	2013
Nikolic [63]	Continuous	Offline	Based	Proposed a non-parametric calibration method		2016

^1^ Optimization: Continuous-time based or discrete-time based; ^2^ Estimator: Whether the calibration is online or offline. ^3^ Target: Whether artificial targets are required.

**Table 4:** Comparison of the advantages and disadvantages of four methods of camera–IMU calibration.

Methods	Principles	Advantages	Disadvantages	References
Decoupling-based	Independently estimate the rotation and translation parameters	Independent estimation of rotation and translation reduces the accumulation of coupled errors	Limited generalizability; high sensitivity to environmental conditions	[39,43]
Filter-based	Use a filtering approach to recursively estimate the states of the camera and IMU	High computational efficiency; suitable for online real-time calibration	Dependent on accurate assumptions about the noise distribution; sensitive to noise models and parameters	[44,47,50]
Optimization-based	Establish a mathematical model with error functions and minimize measurement errors using optimization algorithms	High accuracy with the capability to simultaneously optimize multiple error terms	High computational complexity; poor real-time performance	[58,59,60]
Learning-based	Learn patterns in sensor data from large-scale datasets through data-driven methods	No need for manual feature; strong adaptability to dynamic environments	Dependent on large-scale data; not suitable for unseen scenarios.	[64,73]

**Table 5:** Calibration performance of several camera–IMU methods.

Methods	Translation Absolute Error (mm)				Rotation Absolute Error (°)				Target ^1^	Estimator ^2^	Convergence Time (s)	Robustness
	Mean	x	y	z	Mean	Roll	Pitch	Yaw				
Kelly [47]	-	3.6	−0.1	5.7	-	0.19	0.12	−0.14	Free	Online	50	☆☆☆☆
	-	−0.1	−2.8	−3.2	-	−0.02	−0.06	0.05	Based	Offline	-	
Zachariah [49]	-	0.466	−1.727	1.415	-	0.066	−0.018	0.010	Based	Online	-	☆☆☆
Lee [52]	-	1.7	1.3	1.0	-	0	0	0	Based	Offline	20–30	☆☆☆
Yang [55]	<2	-	-	-	<1	-	-	-	Free	Online	>10	☆☆
Qiu [57]	-	4.8	3.7	3.6	-	-	-	-	Based	Offline	-	☆
Huang [59]	<5	-	-	-	<0.6	-	-	-	Free	Online	>25	☆☆
Fleps [60]	-	0.4	3.0	0	-	−0.3	+0.9	−0.8	Free	Offline	10	☆☆☆☆
Furgale [33]	-	0.73	−0.18	0.02	-	0.001	−0.010	0	Based	Offline	<300	☆☆☆☆

^1^ Target: Whether artificial targets are required; ^2^ Estimator: Whether the calibration is online or offline.

**Table 6:** Calibration performance of several LiDAR–IMU methods.

Methods	Translation Absolute Error (mm)				Rotation Absolute Error (°)				Estimator ^1^	Convergence Time (s)	Robustness
	Mean	x	y	z	Mean	Roll	Pitch	Yaw			
Xia [89]	[1.3, 17.2]	-	-	-	[0.89, 1.22°]	-	-	-	Offline	-	☆☆
Gentil [90]	1	-	-	-	0.01	-	-	-	Offline	-	☆☆☆
Li [92]	-	2.6	1.7	4.2	-	0.366	0.014	0.019	Online	-	☆☆☆
Zhu [93]	6.4	-	-	-	0.4019	-	-	-	Online	10.2	☆☆☆
Yang [94]	-	-	-	-	-	0.427	−0.149	0.282	Online	-	☆☆☆
LV [100]	4.3	-	-	-	0.0224	-	-	-	Offline	25–30	☆☆☆
OA-Calib [101]	4	−1.9	−1.4	−3.2	0.18	0.16	−0.09	0.02	Offline	-	☆☆☆☆
Kim [110]	[21, 125]	-	-	-	[0.501, 0.878]	-	-	-	Online	-	☆☆☆☆
	[27, 36]	-	-	-	[0.847, 0.935]	-	-	-			
	[18, 47]	-	-	-	[0.589, 0.069]	-	-	-			

^1^ Estimator: Whether the calibration is online or offline.

**Table 7:** Characteristics and contributions of representative LiDAR–IMU calibration methods.

References	Optimization ^1^	Estimator ^2^	Contributions	Open-Source	Year
Geiger [86]	Discrete	Offline	Solved the hand–eye calibration problem using the point-to-plane ICP algorithm; constructed a multimodal fusion dataset for autonomous driving		2013
Schneider [88]	Discrete	Online	UKF-based; improved extrinsic parameter estimation in degenerate scenarios by focusing on the rotational component		2018
Gentil [89]	Discrete	Offline	Target-free; employed Gaussian process regression to interpolate IMU measurements		2019
Liu [105]	Discrete	Offline	Based on features such as points or spheres, lines or cylinders, and planes extracted from natural scenes		2020
LI-calib [100]	Continuous	Offline	Continuous-time representation; integrated raw IMU measurement residuals with LiDAR point-to-surface distance residuals	✓	2020
LI-INIT [93]	Discrete	Online	Assessed the degree of excitation in data	✓	2022
OA-Calib [101]	Continuous	Offline	Proposed the information-theoretic metrics to select informative trajectory segments	✓	2022
Wu [102]	Continuous	Offline	Proposed an adaptive frame length LiDAR odometry framework		2023
GRIL-Calib [110]	Discrete	Offline	Proposed ground plane motion (GPM) constraints	✓	2024

^1^ Optimization: Continuous-time based or discrete-time based; ^2^ Estimator: Whether the calibration is online or offline.

**Table 8:** Reviews of the calibration of LiDAR and camera.

Author	Title	Year
Liu [111]	Review of a 3D LiDAR combined with single vision calibration	2021
Li [112]	Automatic targetless LiDAR-camera calibration: A survey	2023
Qiu [113]	External multimodal imaging sensor calibration for sensor fusion: A review	2023
Liao [8]	Deep Learning for Camera Calibration and Beyond: A Survey	2021
Tan [75]	A Review of Deep Learning-Based LiDAR and Camera Extrinsic Calibration	2024
Zhang [114]	3-D LiDAR and Monocular Camera Calibration: A Review	2025

**Table 9:** Camera–LiDAR Calibration Method.

Methods	Advantages	Disadvantages	References
Information theory-based	Independent of scene-specific features; based on clear mathematical principles	Susceptible to local optima; high computational complexity	[115,116]
Feature-based	Directly associates correspondences in physical space	Highly susceptible to environmental factors such as noise and occlusion; high difficulty in cross-modal matching between point clouds and images	[117,118,119]
Ego-motion based	Target-free; no need for initial parameter settings; strong adaptability to dynamic environments	Requires sufficient excitation; susceptible to accumulated errors; demands high-precision time synchronization	[120,121,122]
Learning based	Highly automated; free from manual feature engineering; adaptable to complex scenarios	Requires large-scale datasets; high computational costs; generalization is limited by the training data	[123,124]

**Table 10:** Characteristics and contributions of representative LVI calibration methods.

References	Optimization ^1^	Estimator ^2^	Target ^3^	Contributions	Open-Source	Year
HOU [125]	Discrete	Online	Based	Unified optimization of camera–LiDAR–IMU constraints with global extrinsic refinement		2022
Wang [132]	Discrete	Online	Free	Proposed two-stage initialization for visual-inertial and LiDAR-inertial systems		2022
Multical [128]	Continuous	Offline	Based	Multiple sensors calibration with non-overlapping fields of view	✓	2022
LIU [129]	Continuous	Online	Free	Used natural features for data association; estimated time offset and extrinsic rotation via correlation and hand–eye calibration	✓	2022
Wang [128]	Continuous	Online	Free	Jointly optimized LiDAR point-to-surfel distance error, visual feature reprojection error, and IMU measurement and trajectory derivative errors		2024
Wang [131]	Continuous	Online	Free	Parallel calibration of LiDAR–IMU and camera–IMU extrinsics with convergence criteria and excitation evaluation		2024

^1^ Optimization: Continuous-time based or discrete-time based; ^2^ Estimator: Whether the calibration is online or offline; ^3^ Target: Whether artificial targets are required.

**Table 11:** Calibration performance of several camera–LiDAR–IMU methods.

Methods	Combination ^1^	Translation Absolute Error (mm)				Rotation Absolute Error (°)				Temporal Offset Error (ms)	Estimator ^2^	Target ^3^	Convergence Time (s)	Robustness
		Mean	x	y	z	Mean	Roll	Pitch	Yaw					
Hou [125]	LI	-	2	1	1	-	0.229	0.401	0.286		Online	based	-	☆
	VI	-	3	1	3	-	0.057	0.057	0.343				-	
Zhi [126]	LI	<2.6	-	-	-	0.049	-	-	-		Offline	based	90	☆☆☆
	VI	<2.6	-	-	-	0.032	-	-	-	<0.003			-	
Li [129]	LI	-	2.3	3.6	1.8	-	0.024	0.025	0.014	<0.003	Offline	free	-	☆☆
	VI	-	3.1	3.8	2.7	-	0.027	0.032	0.025	0.06			-	
Li [130]	VI	<0.2	0.16	0.17	0.19	<3	0.04	0.07	0.11	0.09	Offline	free	77	☆☆☆
	I(RS)	<0.2	0.14	0.14	0.2	<3	0.18	0.14	0.09	0.02			-	
Wang [131]	LI	-	[1, 9]	[2, 19]	[1, 34]	-	[0.1, 0.5]	[0.4, 0.5]	[0.9, 1.3]	0.09	Online	free	52.7	☆☆
	VI	-	[0, 18]	[1, 23]	[3, 18]	-	[0.3, 0.9]	[0, 0.3]	[0, 0.8]	-			-	
Wang [132]	VI	-	-	-	-	-	[0.16, 6.81]	[0.62, 4.69]	[0.59, 5.02]	-	Online	free	5.2, 11.9	☆☆
	VI	-	-	-	-	-	0.3	2.9	1.2	-			8~12	
	LI	-	-	-	-	-	1.5	1.3	1.1	3.6			3~5	

^1^ In the combination column, LI, VI, and RS are used to represent LiDAR–IMU, camera–IMU and rolling shutter camera, respectively. ^2^ Estimator: Whether the calibration is online or offline; ^3^ Target: Whether artificial targets are required

**Table 12:** Summary of open-source calibration methods.

References	Sensors ^1^				Spatial	Temporal	Target ^2^	Optimization ^3^	Estimator ^4^	Year
	IMU	Camera	LiDAR	Others						
Geiger [134]		GS	✓		✓		Based	Discrete	Offline	2012
Kalibr [33]	✓	GS, RS			✓	✓	Based	Continuous	Offline	2014
Wang [135]		GS	✓		✓		Based	Discrete	Offline	2017
Liao [138]		GS, RS	✓		✓		Based	Discrete	Offline	2018
VINS-Mono [58]	✓				✓	✓	Free	Discrete	Online	2018
Iyer [124]		GS	✓		✓		Free	DL	Online	2018
Verma [137]		GS	✓		✓		Based	Discrete	Offline	2019
LI-calib [99]	✓		✓		✓		Free	Continuous	Offline	2020
Yuan [138]		GS	✓		✓		Free	DL	Online	2020
Domhof [139]		GS	✓	R	✓		Based	Discrete	Offline	2021
Calirad [140]		GS	✓	R	✓	✓	Based	Continuous	Offline	2021
Tsai [141]		GS	✓		✓		Based	Discrete	Offline	2021
Yuan [142]		GS	✓		✓		Free	Discrete	Offline	2021
Lv [143]		GS	✓		✓		Free	DL	Online	2021
Multical [126]	✓	GS	✓		✓	✓	Based	Continuous	Offline	2022
OA-Calib [101]	✓		✓		✓	✓	Free	Continuous	Offline	2022
LI-init [93]	✓		✓		✓		Free	Discrete	Offline	2022
LVI-ExC [128]	✓	GS	✓		✓	✓	Free	Continuous	Online	2022
Hartzer [50]	✓	GS			✓	✓	Based	Discrete	Online	2022
LIU [125]	✓	GS	✓		✓	✓	Free	Continuous	Offline	2022
Mharolka [144]		GS	✓	T	✓		Free	DL	Online	2022
Duy [145]		GS	✓		✓		Free	DL	Online	2022
Yan [146]		GS	✓		✓		Based	Discrete	Offline	2023
Koide [147]		GS	✓		✓		Free	Discrete	Offline	2023
iKalibr [148]	✓	GS, RS	✓	R	✓	✓	Free	Continuous	Offline	2025

^1^ In the sensor column. I, L, GS, RS, T, and R are used to represent IMU, LiDAR, GS/RS/thermal camera, and radar, respectively. ^2^ Target: Whether artificial targets are required; ^3^ Optimization: Continuous-time based, discrete-time based, or learning-based; ^4^ Estimator: Whether the calibration is online or offline.

**Table 13:** Open datasets summary.

Dataset	Address	Collection Platform	LiDAR	Camera	IMU	Year
KITTI [86]	https://www.cvlibs.net/datasets/kitti/ (accessed on 8 April 2025)	Vehicle	✓	✓		2012
EuRoC [149]	https://projects.asl.ethz.ch/datasets/doku.php?id=kmavvisualinertialdatasets (accessed on 8 April 2025)	UAV		✓	✓	2016
Argoverse 2 [150]	https://www.argoverse.org/av2.html (accessed on 8 April 2025)	Vehicle	✓	✓		
KAIST Urban [151]	https://sites.google.com/view/complex-urban-dataset/home (accessed on 8 April 2025)	Vehicle	✓	✓		
TUM GS-RS [152]	https://github.com/tum-traffic-dataset/tum-traffic-dataset-dev-kit (accessed on 8 April 2025)	Vehicle	✓	✓		2022
TUM-VI [153]	https://cvg.cit.tum.de/data/datasets/visual-inertial-dataset (accessed on 8 April 2025)	UAV		✓	✓	2018
UZH [154]	https://fpv.ifi.uzh.ch/ (accessed on 8 April 2025)	UAV		✓	✓	2019
Oxford Robotcar [155]	https://robotcar-dataset.robots.ox.ac.uk/ (accessed on 8 April 2025)	Vehicle	✓	✓		2017
NCLT [156]	https://robots.engin.umich.edu/nclt/ (accessed on 10 April2025)	Vehicle	✓	✓	✓	2016
4Seasons [157]	https://cvg.cit.tum.de/data/datasets/4seasons-dataset (accessed on 10 April 2025)	Vehicle		✓	✓	2021
Newer College [158]	https://ori-drs.github.io/newer-college-dataset/ (accessed on 10 April 2025)	Vehicle	✓	✓	✓	2020
nuScenes [159]	https://www.nuscenes.org/ (accessed on 10 April 2025)	Vehicle	✓	✓	✓	2019
S3E [160]	https://github.com/chengwei920412/S3E-dataset (accessed on 10 April 2025)	Mobile robot	✓	✓	✓	2024
MUN-FRL [161]	https://mun-frl-vil-dataset.readthedocs.io/en/latest/ (accessed on 10 April 2025)	UAV	✓	✓	✓	2023
Erfoud [162]	https://sites.laas.fr/projects/erfoud-dataset/sites.laas.fr/projects/erfoud-dataset/index.html (accessed on 10 April 2025)	Mobile robot	✓	✓		2020
Urbanloco [163]	https://github.com/weisongwen/UrbanLoco (accessed on 10 April 2025)	Vehicle	✓	✓	✓	2020
UtbmRobotcar [164]	https://epan-utbm.github.io/utbm_robocar_dataset/ (accessed on 10 April 2025)	Vehicle	✓	✓	✓	2020
M2DGR [165]	https://github.com/SJTU-ViSYS/M2DGR (accessed on 15 April 2025)	Mobile robot	✓	✓	✓	2021
NTU-VIRAL [166]	https://ntu-aris.github.io/ntu_viral_dataset/ (accessed on 15 April 2025)	UAV	✓	✓	✓	2022
Hilti-Oxford [167]	https://hilti-challenge.com/dataset-2022.html (accessed on 15 April 2025)	Mobile robot	✓	✓	✓	2023
MCD [168]	https://mcdviral.github.io/ (accessed on 15 April 2025)	Handheld	✓	✓	✓	2024
UrbanNav [169]	https://github.com/IPNL-POLYU/UrbanNavDataset (accessed on 15 April 2025)	Vehicle	✓	✓	✓	2023
A*3D [170]	https://github.com/I2RDL2/ASTAR-3D (accessed on 15 April 2025)	Vehicle	✓	✓		2020
CADC [171]	https://gitee.com/yangmissionyang/cadc_devkit (accessed on 15 April 2025)	Vehicle	✓	✓		2020
Ford Multi-AV [172]	https://avdata.ford.com/downloads/default.aspx (accessed on 15 April 2025)	Vehicle	✓	✓	✓	2020
USVinland [173]	https://github.com/ORCA-Uboat/USVInland-Dataset (accessed on 15 April 2025)	USV	✓	✓		2021

## Data Availability

No new data were created or analyzed in this study. Data sharing is not applicable to this article.
